# Implementation of cardiovascular disease risk prediction scores in Sub-Saharan Africa: a scoping review

**DOI:** 10.1186/s12913-026-15378-1

**Published:** 2026-08-19

**Authors:** Mary Gouws, Naemi Araya, Elinor Harriss, Thomas A. Gaziano, Mike English, Claire M. Keene

**Affiliations:** 1 Health Systems Collaborative, Centre for Global Health and Tropical Medicine, Nuffield Department of Medicine, University of Oxford, Oxford, UK; 2 Cambridge Healthcare Research, London, UK; 3 Bodleian Health Care Libraries, University of Oxford, Oxford, UK; 4 Brigham and Women’s Hospital, Cardiovascular Medicine, Boston, USA; 5 Harvard T.H. Chan School of Public Health, Harvard University, Boston, USA; 6 KEMRI-Wellcome Programme, Nairobi, Kenya; 7 Centre for Infectious Disease Epidemiology and Research, School of Public Health and Family Medicine, Faculty of Health Sciences, University of Cape Town, Cape Town, South Africa

**Keywords:** Cardiovascular disease, Risk prediction, Sub-Saharan Africa, Scoping Review, Implementation

## Abstract

**Background:**

Cardiovascular disease (CVD) risk prediction scores are widely recommended to guide primary prevention, yet their use in routine care across sub-Saharan Africa (SSA) remains limited. As the number of available scores continues to increase, understanding how they are implemented is important in closing the gap between policy and practice. This scoping review aims to map and synthesise the available literature on the implementation of CVD risk prediction scores in SSA.

**Methods:**

This review was conducted in accordance with JBI methodology. MEDLINE, Embase, Global Health (Ovid), CINAHL, Scopus and ProQuest Dissertations and Theses Global were searched. An Implementation Research Logic Model was used to structure data charting and synthesis across implementation determinants, strategies and outcomes.

**Results:**

Of the 4930 publications screened, 41 were included, representing 34 unique evidence sources across ten countries. Four use cases for CVD risk prediction scores were identified: (1) economic and policy considerations; (2) screening for high CVD risk and referral; (3) communication of risk and (4) treatment of high-risk individuals. The most frequently reported barriers to the use of risk scores related to health system and organisational challenges. Implementation strategies primarily focused on task-shifting, training, and short-term material or technological support for community health workers, whereas few studies evaluated strategies to support the uptake of CVD risk scores in health facilities. While feasibility and acceptability outcomes were widely studied, evidence on long-term sustainability of CVD risk scores was limited.

**Conclusion:**

The use of an Implementation Research Logic Model enabled structured synthesis of a heterogeneous and under-theorised evidence base, revealing a mismatch between the breadth of implementation determinants and the narrow range of strategies used to address them. The potential of CVD risk prediction scores, and other clinical prediction tools, to strengthen preventive care in SSA is unlikely to be realised without effective integration into routine health services. Therefore, moving beyond short-term, task-shifted screening pilots toward sustainable implementation approaches addressing the complete CVD care pathway will be essential to ensure that both existing and emerging risk prediction tools translate into equitable improvements in cardiovascular outcomes.

**Supplementary Information:**

The online version contains supplementary material available at https://doi.org/10.1186/s12913-026-15378-1.

## Introduction

Cardiovascular disease (CVD) is a leading cause of morbidity and mortality globally, with a substantial burden in low- and middle-income countries [1, 2]. In sub-Saharan Africa (SSA), deaths due to CVD have increased by more than 50% over the past three decades, and now account for over a million deaths annually in the region [1, 3].

While communicable diseases have historically driven funding, research and service delivery priorities in SSA, the ongoing epidemiological transition, characterised by rising prevalence of hypertension, diabetes, and other CVD risk factors, places growing pressure on health services originally designed to address infectious diseases [3, 4].

In response, policies and clinical guidelines increasingly recommend a total CVD risk approach to primary prevention, rather than management based on individual risk factors alone [5–7]. CVD risk prediction models estimate an individual’s probability of experiencing an atherosclerotic cardiovascular event (heart attack or stroke) over a defined time horizon, typically 5 or 10 years, by combining multiple risk factors such as age, sex, blood pressure, smoking status, diabetes, and lipid levels [8, 9]. These models are operationalised as simplified risk scores to support decision-making in clinical practice, often through charts or electronic tools that guide initiation of preventive therapies at defined risk thresholds.

Well-known risk scores include the Framingham score [10]; the World Health Organisation/International Society of Hypertension (WHO/ISH) 2007 [11] and 2019 [12] scores; and the European Society of Cardiology’s Systematic COronary Risk Evaluation 2 (SCORE-2) [7]. These represent only a small subset of the available models, as the number of CVD risk prediction scores has expanded substantially over the past two decades. A 2016 systematic review identified over 300 published CVD risk prediction models [13] and this number has continued to grow in recent years with the rapid expansion of artificial intelligence and machine-learning approaches [14, 15]. However, only a relatively small proportion of existing scores are operationalised as tools to support clinical decision-making at the point of care [9].

Evidence suggests that even where scores and tools are available, they are often not routinely used in clinical practice and as a result, evidence on their clinical effectiveness remains uncertain [16–19]. A 2017 Cochrane review found that use of CVD risk scores in clinical care to inform clinician decision-making resulted in small reductions in cholesterol, blood pressure and composite CVD risk, and increased prescribing of lipid-lowering medications among higher-risk individuals [19]. However, these estimates were imprecise, with the overall quality of evidence rated as low [19]. This was largely due to heterogeneity in how risk scores were implemented, reliance on short-term and process-oriented outcomes, and a lack of adequately powered studies assessing real-world clinical endpoints [19].

This highlights a disconnect between ongoing investment in the development of increasingly sophisticated risk prediction models and their uptake in clinical practice. Greater attention to the broader clinical, organisational and health service factors influencing use will be essential to realise the potential of both existing and emerging risk prediction tools.

To examine the determinants of CVD risk score implementation in primary care, Muthee et al., (2020) conducted a mixed-methods systematic review guided by the Consolidated Framework for Implementation Research (CFIR) [16]. This review identified multiple interacting barriers related to the healthcare system and clinical setting, users, and elements of risk scoring tools [16]. However, 20/25 (80%) of the included studies were from high-income countries and none were from SSA [16].

Consequently, a gap in context-specific implementation evidence to inform how CVD risk prediction can be sustainably embedded into care in SSA remains. The aim of this scoping review is to map the literature on the implementation of CVD risk prediction scores in SSA and to synthesise reported implementation determinants, strategies and outcomes related to the use of these scores.

## Methods

### Study design

The literature on CVD risk score implementation in SSA is heterogeneous, spanning diverse study designs, settings and tool modalities. A scoping review was therefore selected to map the breadth of available evidence and capture a wide range of implementation-related insights [20].

The review was conducted in accordance with the methodological frameworks proposed by Arksey and O’Malley [20] and refinements by Levac et al. [21], and followed the Joanna Briggs Institute (JBI) guidance for scoping reviews [22]. Reporting adheres to the Preferred Reporting Items for Systematic Reviews and Meta-Analyses Extension for Scoping Reviews (PRISMA-ScR) (Supplementary Material, Table S1) [23]. The protocol is registered on Open Science Framework [24].

### Eligibility criteria

Eligibility criteria were defined using the Population–Concept–Context (PCC) framework (Table 1). No restrictions on publication date or language were applied.

**Table 1:** Review eligibility (inclusion and exclusion) criteria

PCC Element	Inclusion criteria	Exclusion criteria
Population (geographic scope)	Populations in SSA Multi-country studies with extractable SSA-specific data	Populations exclusively outside SSA or multi-country studies with no extractable SSA-specific data
Population (age)	Adults (≥ 18 years)	Studies exclusively involving children or adolescents
Concept (CVD risk score)	Implementation of any CVD risk prediction score, broadly defined (e.g., risk scores, tools, charts, calculators, algorithms, decision-support)	Studies not involving a CVD risk prediction score
Concept (implementation focus)	Studies reporting on any aspect of implementation, or informing implementation of CVD risk scores, including determinants, strategies, or outcomes	Studies focusing solely on risk estimation, model development, validation, or population-level risk quantification with no assessment of implementation
Context (health system level)	Any level of the health system, including community, primary care, secondary, and tertiary settings	None
Context (tool users)	Any user of the risk prediction tool, including healthcare professionals, community health workers, patients, policy-makers	None
Types of evidence sources	Primary research of any design (interventional or observational; quantitative, qualitative, or mixed methods, economic evaluations)	Editorials, commentaries, opinion pieces, narrative syntheses, secondary evidence syntheses
Publication type	Peer-reviewed articles, dissertations and theses, reports, and conference or poster abstracts (where sufficient implementation information was available)	Publications lacking primary data or insufficient information to assess implementation

CVD: cardiovascular disease | SSA: sub-Saharan Africa

### Search strategy

MEDLINE (PubMed), EMBASE (Ovid), Global Health (Ovid), CINAHL (EBSCOhost), SCOPUS and ProQuest Dissertations and Theses Global were searched from inception to the search date (3 September 2025). Search strategies were developed and refined in consultation with a research librarian, EH. The full MEDLINE search strategy is provided in the Supplementary Materials, Table S2.

To maximise sensitivity, the search strategy did not include implementation-specific terms. This decision reflects the inconsistent use of implementation terminology in the literature, where studies describing real-world use of CVD risk scores may not be explicitly framed using implementation science language.

### Evidence selection

All records were imported into EndNote (version 21.5) for de-duplication and subsequently uploaded into Rayyan software [25] for screening. Title and abstract screening was conducted by MG, with a random sample of 20% screened by a second reviewer (NA) for consistency. All eligible full texts were screened by MG and a second reviewer (NA). Inter-rater agreement was assessed in the subset of papers screened by both reviewers. Prior to consensus discussions, inter-reviewer agreement was substantial (Gwet’s AC1 = 0.98) [26].

Forward and backward citation searching of included texts was conducted to identify additional evidence sources. Reasons for exclusion were recorded and summarised in the PRISMA-ScR flow diagram. Non-English records were translated using Google Translate to support screening.

### Data charting and analytic framework

Data were charted using a tool developed and iterated by MG. Key characteristics of the publications (study country and setting, year of publication, study design) and reported use cases of CVD risk prediction scores were extracted.

To organise and synthesise the heterogeneous body of evidence, while ensuring diverse aspects of implementation were included, an Implementation Research Logic Model (IRLM) was used to structure the extraction tool [27]. The IRLM provides a comprehensive approach for organising key components of implementation, including determinants, implementation strategies, mechanisms and outcomes, within a single conceptual framework. Its use enabled consistent categorisation and integration of findings across studies with varying designs and levels of reporting. Determinants were coded according to the Consolidated Framework for Implementation Research (CFIR) domains [28], implementation strategies were charted using the Expert Recommendations for Implementing Change (ERIC) taxonomy [29, 30], and outcomes were categorised according to Proctor’s framework [31].

The IRLM was adapted for this review. Mechanisms linking implementation strategies to outcomes were not analysed, reflecting limited reporting of mechanisms in the included studies and the scoping review’s aim to map existing evidence rather than evaluate causal pathways. The IRLM was therefore used as an organising and synthesis framework rather than a tool for causal inference.

### Methodological quality appraisal

As per the guidance on conducting scoping reviews, we did not appraise the methodological quality or risk of bias of included articles.

## Results

### Search results

**Fig. 1:**
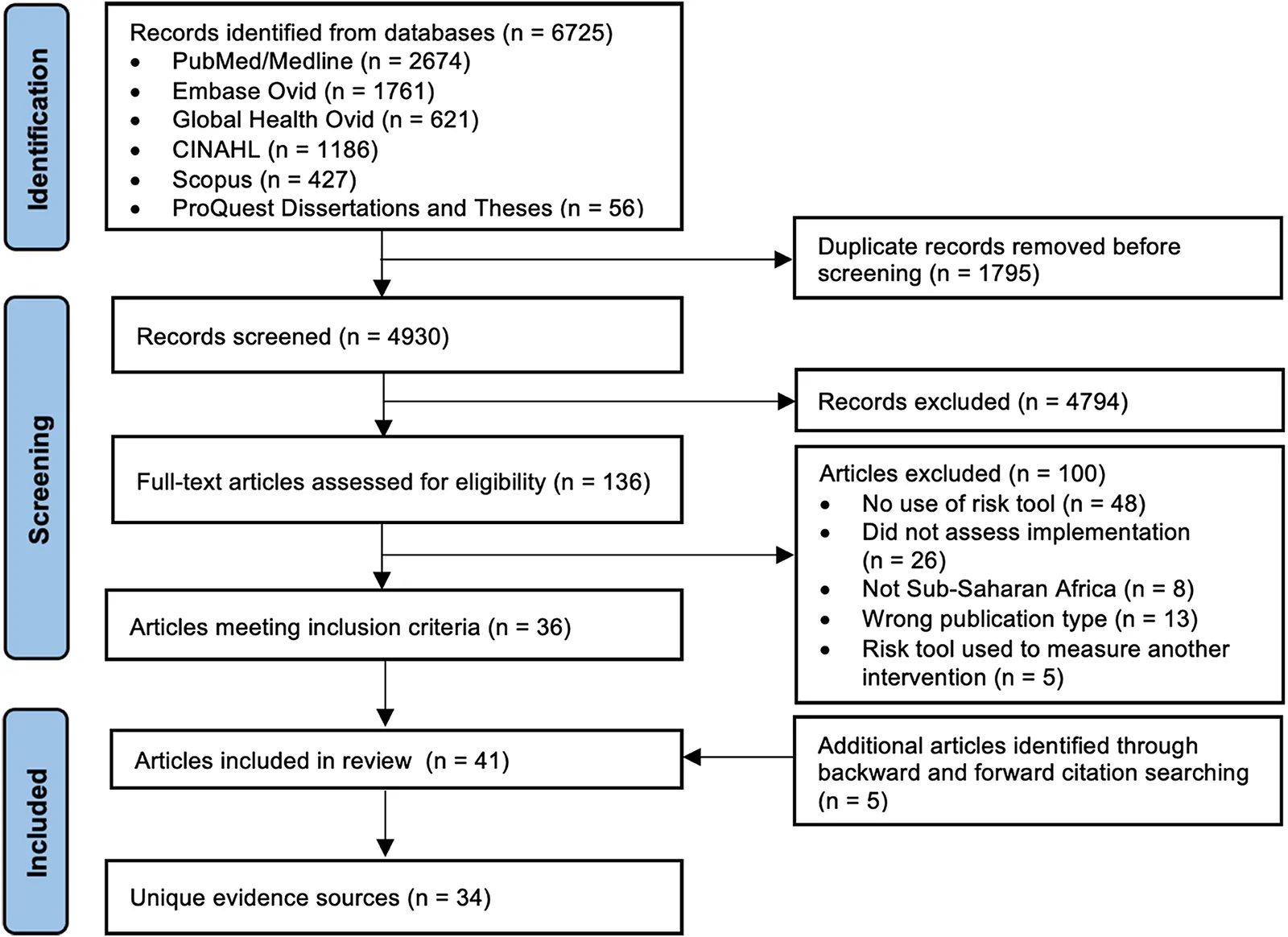
Preferred reporting items for systematic reviews and meta-analyses extension for scoping reviews (PRISMA-ScR) flow diagram showing study screening and selection

4,930 records were screened at title and abstract stage, and 136 articles underwent full-text review (Fig. 1). Following full-text screening, 36 publications met the inclusion criteria. An additional 5 publications were identified through backward and forward citation searching, giving a total of 41 publications. During data charting, some publications were found to report on the same underlying evidence source, such as the same study, programme, or implementation effort. These were grouped and treated as a single evidence source to avoid over-representation and to improve clarity of synthesis. Therefore, the evidence represented 34 unique evidence sources.

### Included studies

**Fig. 2:**
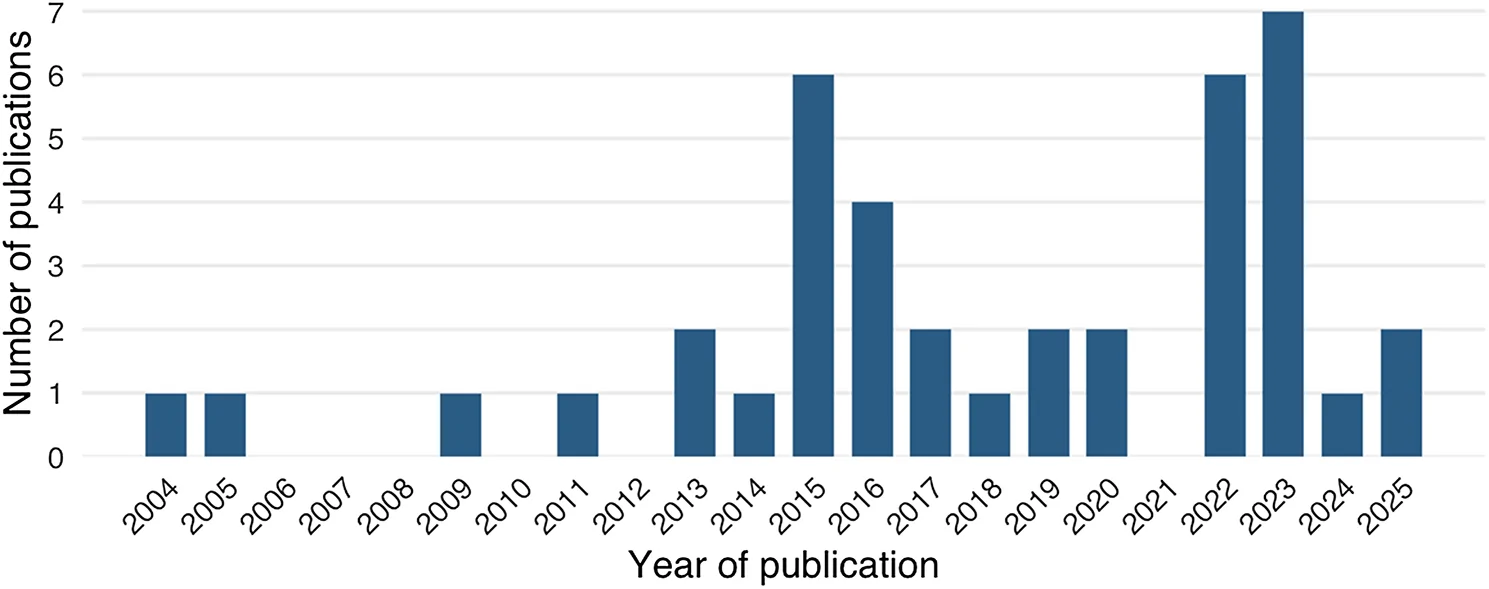
Distribution of publications by year (*n* = 41)

The key characteristics of included studies are provided in Table S3, Supplementary Materials. The number of publications increased from 2004 to 2025 (*n* = 41), although no publications were identified in 2021 (Fig. 2). A higher number of publications in 2015–2016 reflects six outputs from a study assessing a task-shifting initiative to community health workers (CHWs) in South Africa [32–37]. Ten SSA countries were represented in the 34 distinct evidence sources, including South Africa (*n* = 7), Nigeria (*n* = 7), Kenya (*n* = 6), Ethiopia (*n* = 5), Uganda (*n* = 2), Seychelles (*n* = 2), Rwanda (*n* = 2), Tanzania (*n* = 2), Ghana (*n* = 1) and Botswana (*n* = 1).

The evidence base included 5 clinical audits [17, 38–41]; 7 cross-sectional surveys of clinicians and community members [18, 42–47]; 5 qualitative studies examining perceptions of risk and tool acceptability [45, 48–51]; 11 mixed-methods and operational implementation pilot studies [32–34, 52–59]; 12 economic evaluations and modelling studies assessing cost-effectiveness or policy scenarios [36, 60–70] and one randomised controlled trial [42]. Across evidence sources, implementation was variably positioned: in some studies, implementation questions were the primary focus (e.g., feasibility, acceptability, or referral completion), while in others implementation findings were incidental, emerging from audits of practice gaps, surveys of awareness, or economic models that assumed delivery conditions and uptake.

**Fig. 3:**
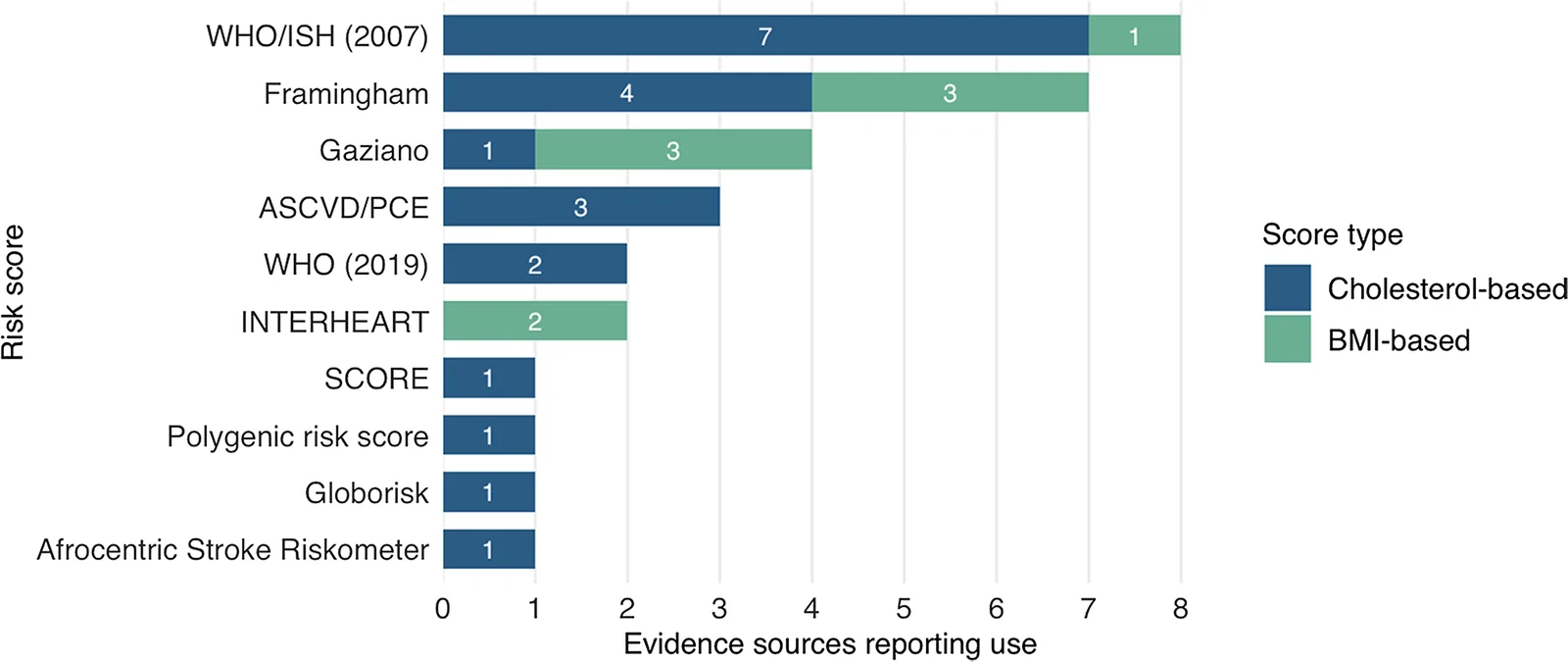
Distribution of cardiovascular disease risk prediction scores reported across distinct evidence sources. Six evidence sources did not report a named risk prediction score (e.g., studies of cardiovascular risk communication and retrospective audits assessing whether any risk score had been documented in clinical records) and are therefore not shown. *Gaziano score*: Risk score developed by Gaziano et al., using the NHANES cohort in the United States [71]. *Polygenic risk score*: Refers to a theoretical precision-medicine risk score explored in a survey of South African clinicians’ perspectives [46]. The *Afrocentric Stroke Riskometer*: Developed using case-control data and piloted in Nigeria and Ghana [72]. WHO: World Health Organisation | ISH: International Society of Hypertension | PCE: Pooled Cohort Equations | SCORE: Systematic Coronary Risk Evaluation

A range of well-known risk prediction scores were reported (Fig. 3). The majority were cholesterol-based (laboratory-based) scores, requiring a blood test to measure lipid levels for risk calculation. BMI-based versions (also called non-laboratory or office-based scores), which replace cholesterol with body mass index, were less frequently used. The WHO/ISH (2007) [11] scores were the most frequently applied, reported in eight evidence sources, with most using cholesterol-based approaches (*n* = 7), and only one application of a BMI-based version.

### IRLM for CVD risk prediction scores in SSA

**Fig. 4:**
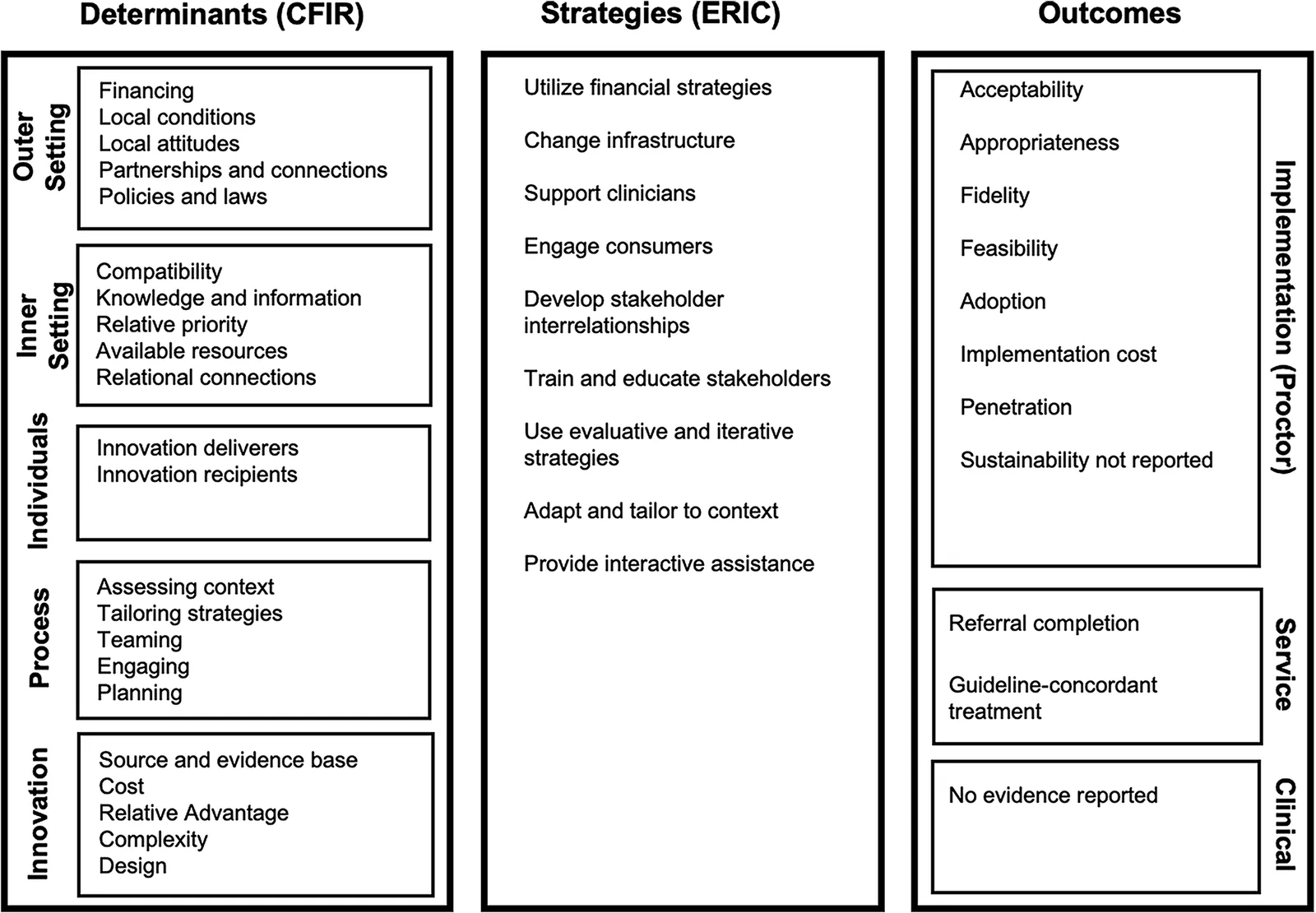
Adapted Implementation Research Logic Model (IRLM) showing categories of determinants, strategies and outcomes of implementation of cardiovascular disease risk prediction scores in Sub-Saharan Africa. Mechanisms were not reported in the available literature. CFIR: Consolidated Framework for Implementation Research | ERIC: Expert Recommendations for Implementing Change

Implementation determinants, strategies, and outcomes were mapped using an adapted IRLM [27] (Fig. 4; Supplementary Figure S1).

Determinants were reported across all CFIR domains [28] (Fig. 5A). Compared with the breadth of determinants, the range of implementation strategies (Fig. 5B) described in the literature was relatively narrow, with task-shifting of CVD risk screening to CHWs being the most common strategy, typically supported by training and supervision [34, 37, 52–54, 56, 58].

**Fig. 5A:**
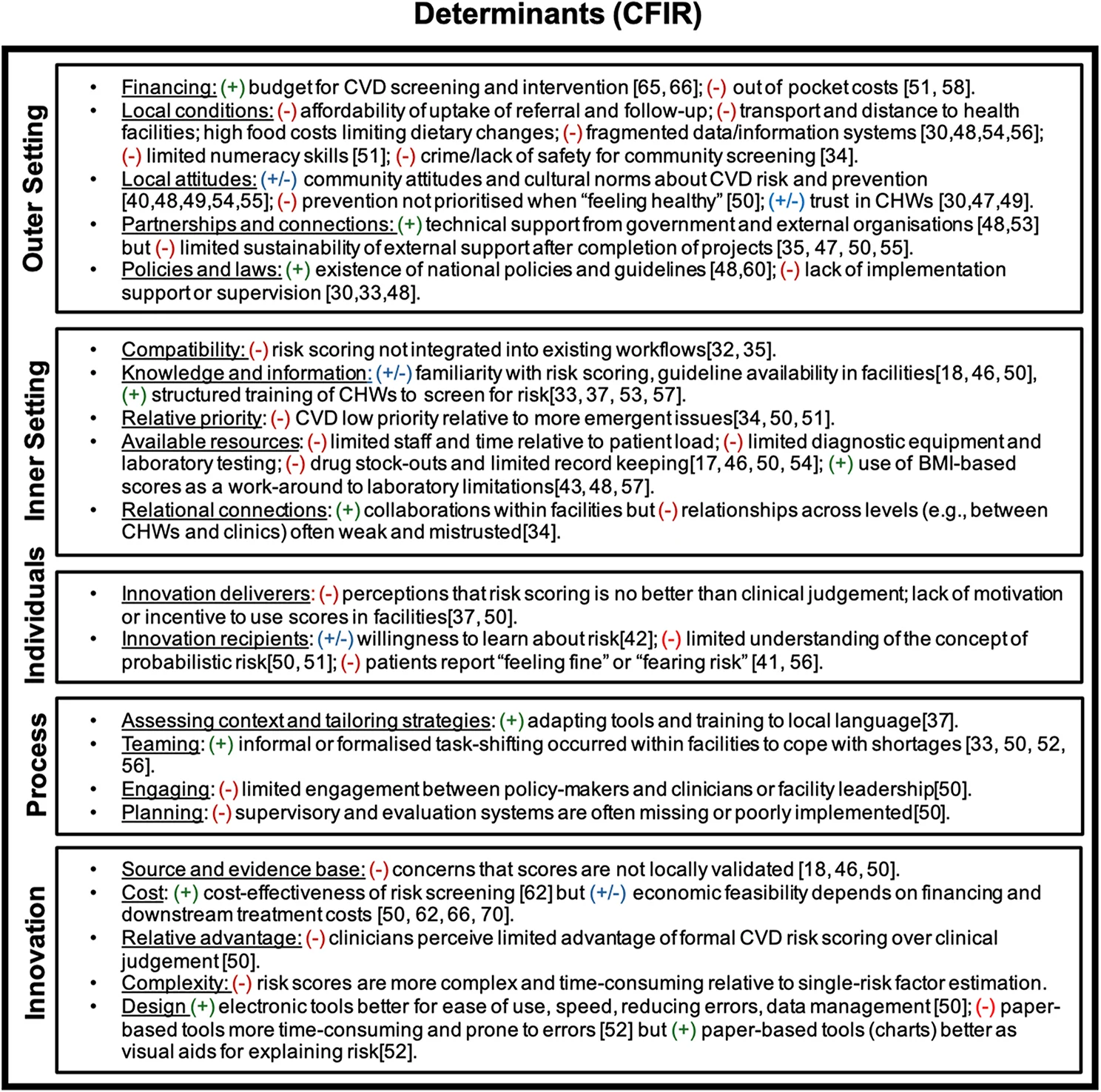
Determinants of implementation of cardiovascular disease risk prediction scores in Sub-Saharan Africa, categorised using the Consolidated Framework for Implementation Research (CFIR). Key: (+) facilitator; (-) barrier; (+/-) facilitator or barrier depending on context. CVD: Cardiovascular Disease | CHW: Community Health Worker

Implementation outcomes were variably reported (Fig. 5C). Acceptability was generally high among trained CHWs, whereas patient acceptability varied according to perceptions of risk, financial barriers to follow-up, and trust in providers [42, 50]. Adoption of CVD risk scoring in routine clinic or hospital care was limited, with several studies reporting low or absent use of risk scores in clinical consultations [38, 41, 50]. Penetration of guideline-concordant risk-based treatment was low and sustainability was not assessed in any of the studies [17, 38]. Mechanisms linking strategies to outcomes were not explicitly reported in the available literature.

**Fig. 5B:**
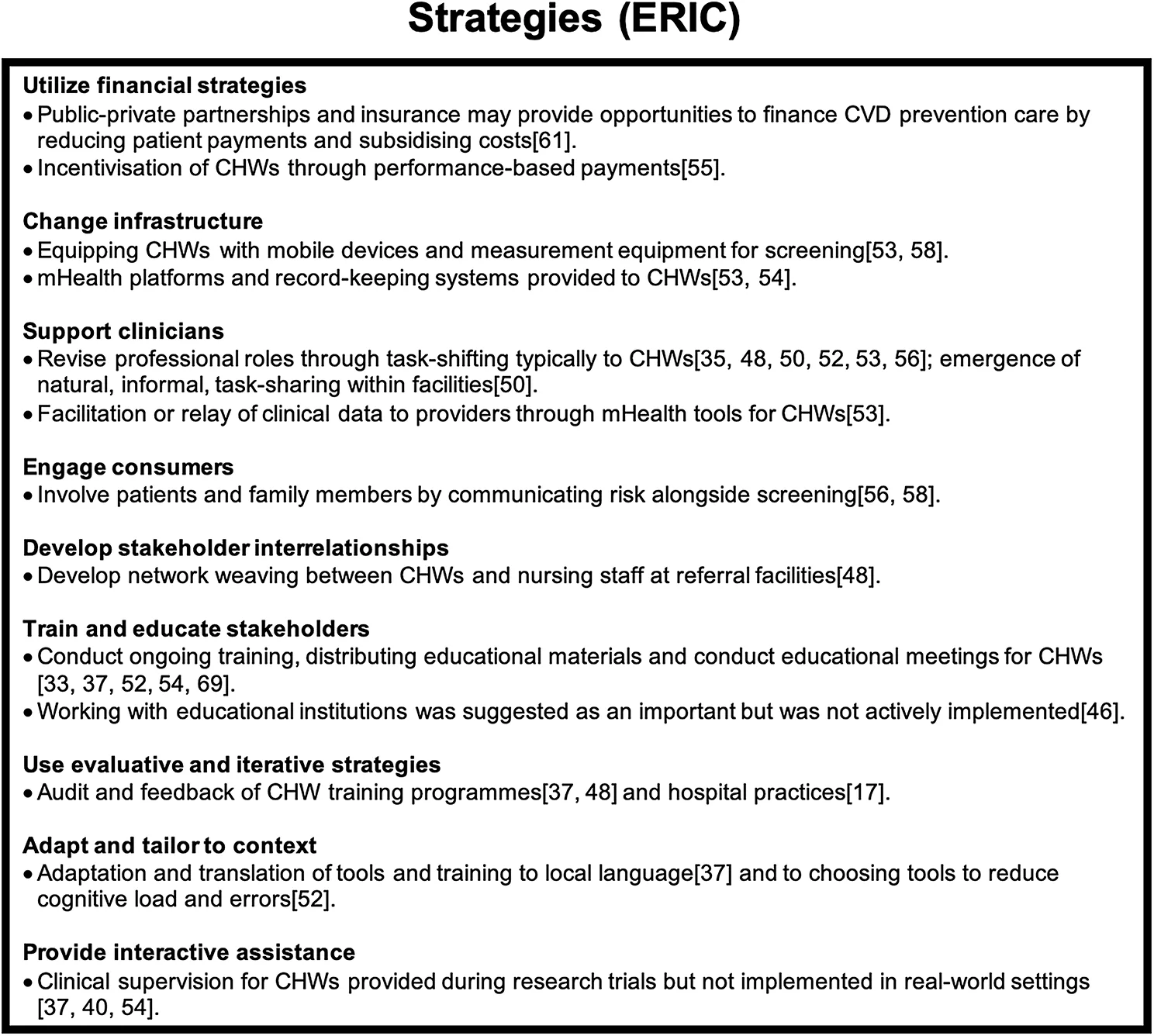
Strategies used in the implementation of cardiovascular disease risk prediction scores in Sub-Saharan Africa, grouped using the Expert Recommendations for Implementing Change framework (ERIC). CVD: Cardiovascular Disease | CHW: Community Health Worker

**Fig. 5C:**
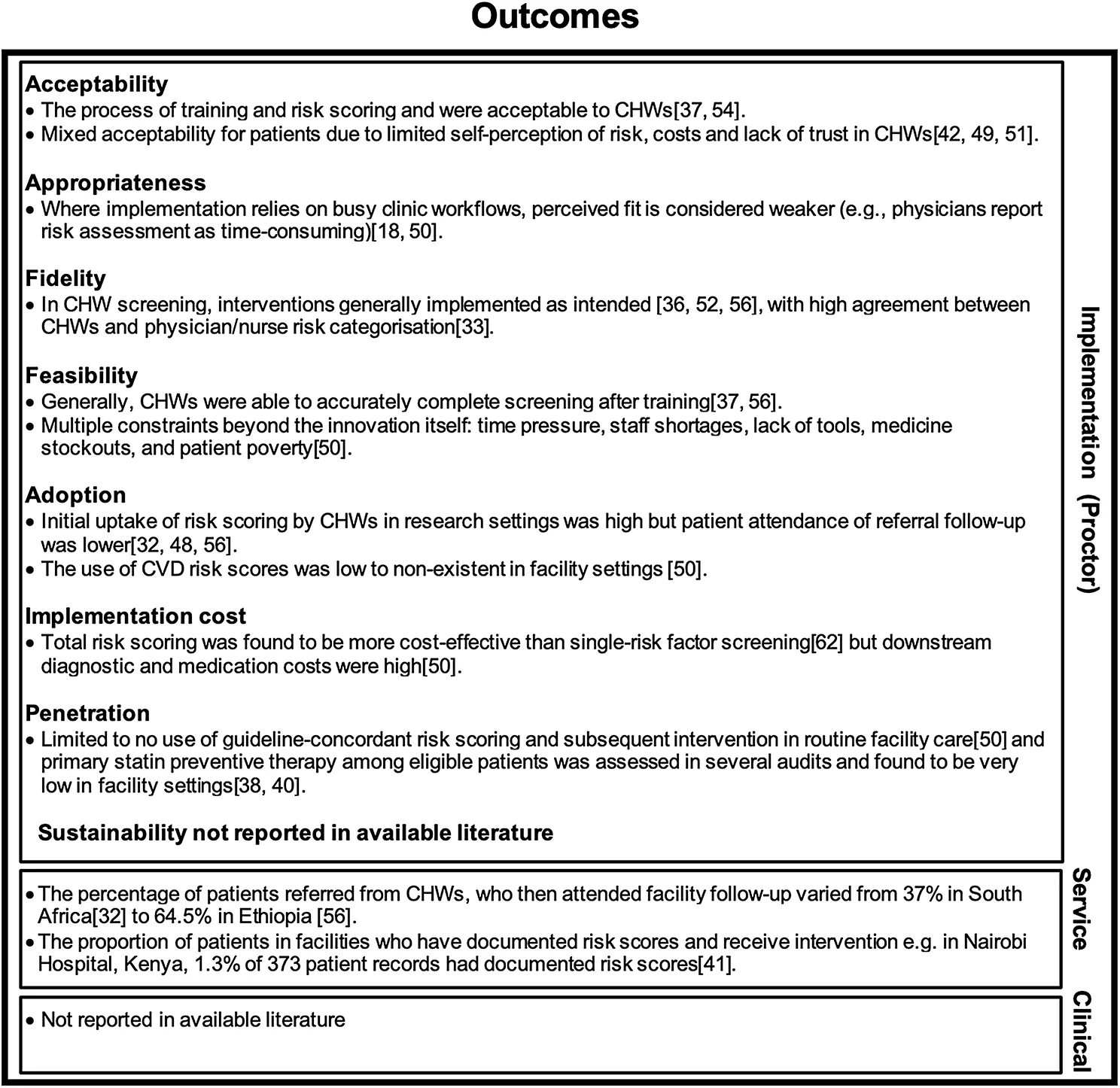
Outcomes of implementation of cardiovascular disease risk prediction scores in Sub-Saharan Africa. CVD: Cardiovascular Disease | CHW: Community Health Worker

### Use cases for CVD risk prediction scores

**Fig. 6:**
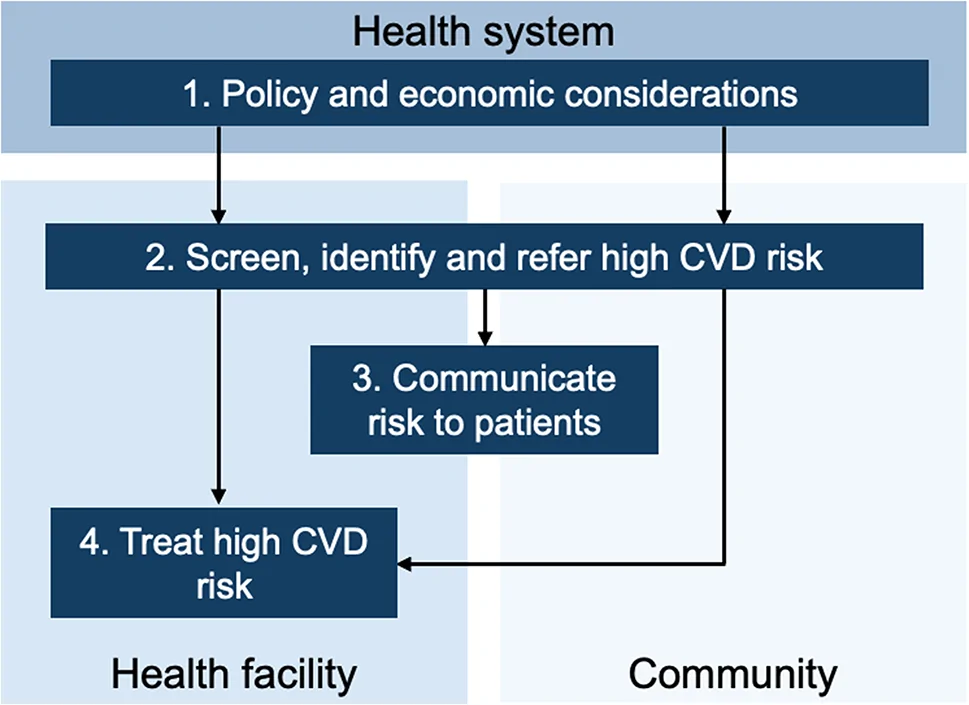
Use cases for cardiovascular disease (CVD) risk prediction scores identified through the review: (1) Policy and economic considerations, (2) Screening, identifying and referring for high CVD risk, (3) Communicating risk to patients, and (4) Treating high CVD risk

Mapping determinants, strategies, and outcomes highlighted that implementation of CVD risk prediction scores occurred across several operational contexts. These contexts differed in terms of who used the risk scores, where they were applied and the purpose of risk estimation. We identified four main use cases for CVD risk prediction scores in SSA: (1) Policy and economic considerations at a health system level; (2) Screening, identification and referral of patients at high CVD risk; (3) Communicating risk to patients and (4) Supporting treatment decisions for individuals identified as high risk (Fig. 6).

#### Policy and economic considerations

Multiple economic evaluations demonstrated that using absolute CVD risk to guide preventive treatment can improve cost-effectiveness and efficiency compared to single-risk factor approaches, by targeting preventive pharmacotherapy to individuals at high absolute risk [60, 62–68]. However, the strength of the cost-effectiveness conclusions depended heavily on local context and assumptions, where diagnostic tests, medications and associated service delivery costs were the major cost drivers [60, 61, 67, 70].

Furthermore, even where interventions appeared cost-effective at a population level, the downstream costs of providing long-term treatment following screening could remain unaffordable in resource-limited health systems [61, 70], and budget-impact analyses were rarely conducted in the included studies. Although one study in rural Nigeria [61] proposed public–private insurance or partnership models as a potential strategy to enable financing of CVD prevention care, explicit financing strategies to support routine implementation of risk-based treatment were largely absent from the literature.

#### Screening, identification and referral of high CVD risk

Seven evidence sources (14 publications) involved training CHWs to identify individuals at elevated CVD risk, followed by initiation of referral to health facilities [32, 34, 35, 52–58]. Across these studies, CHWs were trained to use cholesterol-based scores, often supported by simplified protocols or mHealth applications, to identify individuals at elevated CVD risk and initiate referral. CHWs were generally capable of accurately completing risk score calculations, with low error rates assessed in South Africa [33, 52] and Rwanda [58].

While screening rates were relatively high, referral completion varied widely by setting. In one South African follow-up study of people identified “at high-risk” during screening, only 37% attended a clinic visit [32]. In Ethiopia, 80/124 (64.5%) screened participants voluntarily attended a local clinic for further assessment and management within four weeks of risk screening [56]. Reasons for non-attendance were similar across settings, and included financial barriers, participants being too busy, finding follow-up inconvenient, “feeling fine” and a lack of trust in CHWs [32, 56, 58]. No studies assessed longer-term penetration or sustainability of programmes involving CHW screening and referral.

Use of risk scores in clinics and hospitals was generally limited [46]. In a survey of 106 doctors across public and private facilities in Port Harcourt in Nigeria, although 70% of doctors surveyed were aware of risk scores, only 28% reported regular use in practice [18]. Muthee (2022) observed 81 consultations between clinicians and patients across seven facilities in Kilifi country, Kenya and did not observe a single use of a CVD risk prediction score [50]. The most frequently cited reasons for not calculating scores were unfamiliarity with how to apply risk scores and the perceived time-consuming nature of calculating the scores in consultations [18]. No studies reported strategies specifically designed to improve uptake of risk scoring in facility settings.

Several publications suggested the integration of CVD risk screening into existing infectious disease programmes, including HIV treatment programmes and tuberculosis (TB) contact screenings [39, 40, 70]. Two audits, one in Nigeria, and one in Botswana, found CVD risk stratification and subsequent prescribing of statins was suboptimal in these settings, though no formal strategies were implemented to enhance screening uptake in the studies [39, 40].

#### Communicating risk to patients

Across multiple settings, participants either could not estimate personal risk or produced estimates that did not align with objectively calculated risk [47–49, 51]. In Seychelles, only half of patients could report an estimate of their CVD risk, and these were frequently underestimations when compared to objectively calculated risk scores [47]. Similarly, in Ethiopia and Nigeria, adults’ self-perceived risks were frequently reported as low relative to calculated risk [42, 45]. Higher socioeconomic status and education were associated with greater ability to accurately report perceived risk, while underestimation of risk was common among younger and less-educated participants [42, 45].

Additionally, familiarity with CVD terminology and individual risk factors did not equate to comprehension of probabilistic or absolute risk [48, 49, 51]. For example, in a low-income, peri-urban setting in South Africa, while participants were familiar with individual CVD risk factors, the concept of risk was perceived in binary terms, making numerical or graded probability of risk communication largely ineffective [51]. As such, quantitative formats (percentages, probabilities, time horizons) were often not interpretable without substantial support, and participants preferred an interactive explanation with categorical formats such as colour-coded categories to indicate low/moderate/high risk [43, 48, 49, 51].

#### Treatment of individuals at high CVD risk

Once a risk estimate has been calculated and communicated, the next step is clinical action, such as initiating blood pressure– or cholesterol-lowering therapy, for individuals identified as high risk.

Across the included studies, evidence on the use of risk scores to guide treatment decisions was limited. In Nairobi Hospital, a private, tertiary hospital in Kenya, a retrospective review of hypertensive patient records found low documentation of formal CVD risk assessment (1.3% of 373 patient records) [41]. Almost half of patients in this study were prescribed statins, despite only 7% being classified as high risk and therefore eligible for statin therapy [41]. Similarly, studies from Ethiopia [38], Botswana [40] and Nigeria [39], demonstrated discordance between statin prescribing and eligibility according to risk profiles. Some of the barriers to using risk scores to guide intervention included clinician perceptions of scores being a waste of time, being no better than clinical judgement, or not appropriately validated for their patient population [18, 50].

## Discussion

While research on CVD risk prediction in SSA has increased over the past decade, evidence of real-world implementation remains fragmented. Although a temporary gap in publications was observed in 2021 (possibly due to disruptions in non-communicable disease research during the COVID-19 pandemic), the evidence base identified in this review has expanded over time. The evidence in SSA remains geographically concentrated, with the majority of studies originating from South Africa, Nigeria, Kenya and Ethiopia.

The findings of this review extend those of the previous mixed-methods systematic review by Muthee et al., (2020) which synthesized determinants of CVD risk score implementation but drew predominantly on evidence from high-income settings [16]. In contrast, this review captures evidence from SSA that was absent from the prior synthesis. This reflects both the increase in SSA-based publications in recent years, as well as our deliberately broad search strategy, which did not rely on implementation-specific terminology and therefore identified a wider range of studies incorporating the use of CVD risk prediction scores.

By using an IRLM as a guiding framework, this review builds on the work of Muthee et al. by moving beyond cataloguing implementation determinants to also synthesise implementation strategies and outcomes. This analytical approach revealed a disconnect between the wide range of reported implementation determinants and the comparatively limited implementation strategies employed and outcomes evaluated.

### Determinants of implementation

Determinants identified in the SSA literature broadly align with those reported by Muthee et al., who presented barriers and facilitators across three domains: the healthcare system and clinical setting, users of the tools, and the tools themselves [16]. In both our review and the Muthee et al. review, implementation is shaped by multiple interacting determinants. However, while Muthee et al. concluded that no single determinant appeared consistently more influential than another [16], our review’s evidence base suggests that within resource-constrained settings in SSA, barriers within the inner and outer setting CFIR domains (health system capacity, referral pathways and resource availability) were the predominant constraints.

This predominance of system-level barriers creates a particular implementation challenge: even when clinician- and tool-level factors facilitate uptake (for example, high acceptability and feasibility of CHW-led screening), broader health system barriers (weak referral pathways, inconsistent access to medications) may undermine their impact. Consequently, implementation determinants in SSA are not fundamentally different in kind from those identified by Muthee et al.; rather, health system barriers could be viewed as bottlenecks that constrain the effectiveness of clinician- and tool-level implementation strategies, and therefore limit uptake of CVD risk scores in routine practice.

### Implementation strategies and outcomes

In contrast to the breadth of identified determinants, the range of implementation strategies reported was narrow and largely centred on task-shifting of CVD risk screening to CHWs, typically supported through training, educational materials, and short-term provision of mHealth-enabled screening tools. Many of the CHW-led studies reported favourable findings on feasibility, acceptability and initial adoption of CVD risk scoring. However, no studies assessed longer-term sustainability beyond the trial period.

One of the major challenges limiting the scale-up of CHW screening and referral initiatives is the limited formalisation and incorporation of CHWs into sustained non-communicable disease services, which remains an ongoing issue in many SSA countries. Therefore, while community-based screening models are technically viable, their translation into long-term service delivery or clinical outcomes remains uncertain [73].

Globally, alternative approaches to integrating risk screening in existing service touchpoints have been explored. For example, in Tasmania, Australia, absolute CVD risk estimation was implemented at pathology collection sites through a self-directed screening station [74]. Comparable approaches have been proposed in SSA, including pharmacy-based CVD risk screening; however, implementation has not been formally evaluated in the published literature [75–77].

In practice, task-shifting of CVD risk scoring to community settings may be well suited to initial screening and identification. However, when risk scores are intended to inform treatment decisions, they should be integrated into routine clinical care and available to the decision-maker within the health facility. Despite the literature identifying numerous barriers to implementing CVD risk scores within health facilities, particularly within the CFIR Inner Setting domain (competing clinical priorities, poor workflow integration, cost of medications), few implementation strategies targeting these barriers were identified. Thus, there is a clear misalignment between the determinants identified in the literature, and the strategies that are currently being employed to enhance implementation.

These implementation gaps also have important implications for economic considerations of CVD risk-based approaches. A substantial body of evidence identified in this review assessed economic feasibility; however, most of these analyses rely on assumptions of high rates of screening, functional referral systems, consistent access to diagnostics and medications. The implementation evidence synthesised in this review suggests such assumptions may be optimistic in many SSA contexts, where routine use of risk scores is low and downstream care pathways are fragmented. For example, an economic evaluation of opportunistic CVD screening among household contacts of people diagnosed with TB found that the intervention was not cost effective, largely due to the downstream costs of providing long-term preventive treatment [70]. Without empirical evaluation of how implementation barriers affect real-world effectiveness and costs, the economic attractiveness of risk-based approaches may be overstated.

### CVD risk scores in the context of Clinical Decision Support

CVD risk scores can be considered as a form of clinical decision support (CDS), and their implementation challenges reflect those observed more broadly across clinical prediction rules and decision support systems. Foundational work by Kawamoto et al. identified four features of CDS associated with improved clinical practice: (1) automatic provision of decision support as part of clinician workflow; (2) provision of recommendations based on assessments; (3) provision of decision support at the time and location of decision making; and (4) computer-based decision support [78]. Across the SSA literature, three of these four features were almost entirely absent: automatic provision of decision support, provision of decision support at the point of clinical decision making and computer-based decision support. Risk scores were most commonly implemented as stand-alone screening tools, particularly by CHWs, with relatively few strategies supporting their routine use within health facilities or directly linking risk estimation to treatment decisions.

The requirements described by Kawamoto et al., are particularly important for long-term preventive risk scores. Unlike acute severity tools used in emergency and critical care, such as the Glasgow Coma Scale or acute sepsis scores, which inform immediate decisions within a single clinical encounter, CVD risk scores depend on longitudinal care pathways, including follow-up, access to treatment, and sustained adherence. As a result, their effectiveness is closely tied to health system integration and continuity of care.

The NHS Health Check programme in the United Kingdom provides a useful comparator for how CVD risk prediction can be operationalised at scale [79]. In this setting, risk assessment is embedded within primary care through electronic health records, automated calculation, and defined referral and treatment pathways, linking risk identification directly to clinical action [79].

While some components of the NHS Health Check model, particularly digital infrastructure, may not be readily transferable to many SSA settings, the underlying principles of integration are useful. These include aligning risk assessment with existing clinical encounters, clearly defining responsibility for acting on risk information, and ensuring linkage between screening, decision-making, and access to treatment. However, the NHS Health Check programme has also experienced challenges, including variable uptake and inequalities in participation, indicating that integration into routine care remains complex even in well-resourced health systems.

### Implications for future research and practice

To translate CVD risk prediction into population health gains in SSA, future research and implementation efforts should move beyond feasibility studies and invest in integrated, sustainable strategies that address key system constraints, including access to affordable medications, workforce capacity and routine data systems.

As the number and complexity of available risk prediction scores continues to expand, with increasing investment in large datasets, and artificial intelligence–driven approaches, implementation should be viewed as a concurrent process if the aim is to translate into improved patient outcomes. Decisions made during model development, such as variable selection, format, interpretability, and linkage to clinical actions, are also implementation decisions. Models that are designed with consideration of how they will be used in practice are more likely to be adopted and sustained. Co-design with clinicians, patients and policy-makers may help to navigate this challenge.

### Strengths and limitations

This scoping review has several strengths. The breadth of the search strategy, conducted without implementation-related search terms, enabled capture of a wide range of relevant studies, with implementation signals considered through the title and abstract screening process. This allowed us to capture SSA-specific findings that complement and triangulate the previous systematic review by Muthee et al., while extending the evidence base. The use of an IRLM provided a structured approach to extracting and synthesising a heterogeneous evidence base and enabled identification of the gaps and misalignment of reported determinants with the strategies employed, and the outcomes assessed.

Several limitations should also be acknowledged. As a scoping review, the findings reflect the published literature and may not fully represent the realities of health service delivery. Additionally, we did not formally assess the methodological quality of included sources or evaluate the extent to which reported measures reflected underlying implementation constructs. While some studies were explicitly designed to evaluate implementation outcomes, others inferred implementation from clinical audits, surveys or routine practice data. Consequently, reported outcomes such as adoption, penetration and acceptability should be interpreted in the context of the underlying study designs.

Although the adapted IRLM provided a useful framework for organising and synthesising the evidence, it was used as an organising rather than causal framework. Combined with the heterogeneity of the included studies, variation in how implementation was conceptualised and measured, and limited reporting of implementation mechanisms, this limits the strength of inferences that can be drawn regarding relationships between implementation determinants, strategies and outcomes.

## Conclusion

The evidence base on CVD risk prediction score implementation in SSA is heterogeneous and remains limited in scope, with most studies focused on feasibility and screening rather than sustained integration into routine care. The review highlights a mismatch between the breadth of implementation determinants identified across the literature and the comparatively narrow range of implementation strategies employed to address them.

While community-based, task-shifted approaches can achieve acceptable and accurate risk screening, their impact is constrained by broader health system and organisational barriers, including weak referral pathways, limited facility-level integration, and inconsistent access to treatment. As a result, implementation successes have largely been confined to early stages of the care pathway and have rarely been sustained beyond research settings.

These findings suggest that CVD risk prediction should not be considered a discrete intervention, but as a component of an integrated care pathway linking risk identification to clinical decision-making and long-term management. Future implementation efforts should therefore move beyond screening-focused interventions towards strategies that are explicitly aligned with system-level constraints, alongside investment in health system integration and access to essential medicines.

## Supplementary Information

Below is the link to the electronic supplementary material.

Supplementary Material 1

## Data Availability

The extracted summary data supporting the findings of this scoping review are provided in the supplementary materials. The full dataset is available from the corresponding author on reasonable request. The study protocol is available online on Open Science Framework.

## Abbreviations

ASCVD: Atherosclerotic cardiovascular disease
BMI: Body mass index
CDS: Clinical decision support
CFIR: Consolidated Framework for Implementation Research
CHW: Community health worker
CVD: Cardiovascular disease
ERIC: Expert Recommendations for Implementing Change
IRLM: Implementation Research Logic Model
ISH: International Society of Hypertension
JBI: Joanna Briggs Institute
LMIC: Low- and middle-income country
MEDLINE: Medical Literature Analysis and Retrieval System Online
mHealth: Mobile health
NHS: National Health Service
PCC: Population–Concept–Context
PRISMA-ScR: Preferred Reporting Items for Systematic Reviews and Meta-Analyses Extension for Scoping Reviews
RCT: Randomised controlled trial
SCORE: Systematic COronary Risk Evaluation
SSA: Sub-Saharan Africa
TB: Tuberculosis
WHO: World Health Organization
