# Supplementary material for: Implementation of cardiovascular disease risk prediction scores in Sub-Saharan Africa: a scoping review
Source: BMC Health Serv Res. 2026 Aug 19;26:1401. doi: 10.1186/s12913-026-15378-1 (PMC13670703; doi:10.1186/s12913-026-15378-1)
Supplement: Supplementary file 1 [file 12913_2026_15378_MOESM1_ESM.docx]

**Supplementary Materials**

**Table S1:** Completed Preferred Reporting Items for Systematic reviews and Meta-Analyses extension for Scoping Reviews (PRISMA-ScR) Checklist.

| **SECTION** | **ITEM** | **PRISMA-ScR CHECKLIST ITEM** | **REPORTED ON PAGE #** |
| --- | --- | --- | --- |
| **TITLE** | | | |
| Title | 1 | Identify the report as a scoping review. | 1 |
| **ABSTRACT** | | | |
| Structured summary | 2 | Provide a structured summary that includes (as applicable): background, objectives, eligibility criteria, sources of evidence, charting methods, results, and conclusions that relate to the review questions and objectives. | 2 |
| **INTRODUCTION** | | | |
| Rationale | 3 | Describe the rationale for the review in the context of what is already known. Explain why the review questions/objectives lend themselves to a scoping review approach. | 4 |
| Objectives | 4 | Provide an explicit statement of the questions and objectives being addressed with reference to their key elements (e.g., population or participants, concepts, and context) or other relevant key elements used to conceptualize the review questions and/or objectives. | 4,5 |
| **METHODS** | | | |
| Protocol and registration | 5 | Indicate whether a review protocol exists; state if and where it can be accessed (e.g., a Web address); and if available, provide registration information, including the registration number. | 6 |
| Eligibility criteria | 6 | Specify characteristics of the sources of evidence used as eligibility criteria (e.g., years considered, language, and publication status), and provide a rationale. | 6,7 |
| Information sources* | 7 | Describe all information sources in the search (e.g., databases with dates of coverage and contact with authors to identify additional sources), as well as the date the most recent search was executed. | 7 |
| Search | 8 | Present the full electronic search strategy for at least 1 database, including any limits used, such that it could be repeated. | Table S2, Supplementary Materials |
| Selection of sources of evidence† | 9 | State the process for selecting sources of evidence (i.e., screening and eligibility) included in the scoping review. | 8 |
| Data charting process‡ | 10 | Describe the methods of charting data from the included sources of evidence (e.g., calibrated forms or forms that have been tested by the team before their use, and whether data charting was done independently or in duplicate) and any processes for obtaining and confirming data from investigators. | 7 |
| Data items | 11 | List and define all variables for which data were sought and any assumptions and simplifications made. | 8 |
| Critical appraisal of individual sources of evidence§ | 12 | If done, provide a rationale for conducting a critical appraisal of included sources of evidence; describe the methods used and how this information was used in any data synthesis (if appropriate). | Not applicable |
| Synthesis of results | 13 | Describe the methods of handling and summarizing the data that were charted. | 8 |
| **RESULTS** | | | |
| Selection of sources of evidence | 14 | Give numbers of sources of evidence screened, assessed for eligibility, and included in the review, with reasons for exclusions at each stage, ideally using a flow diagram. | 9 |
| Characteristics of sources of evidence | 15 | For each source of evidence, present characteristics for which data were charted and provide the citations. | 9, 10, Table S3 Supplementary Materials |
| Critical appraisal within sources of evidence | 16 | If done, present data on critical appraisal of included sources of evidence (see item 12). | Not applicable |
| Results of individual sources of evidence | 17 | For each included source of evidence, present the relevant data that were charted that relate to the review questions and objectives. | Table S3, Supplementary Materials |
| Synthesis of results | 18 | Summarize and/or present the charting results as they relate to the review questions and objectives. | 8-14 |
| **DISCUSSION** | | | |
| Summary of evidence | 19 | Summarize the main results (including an overview of concepts, themes, and types of evidence available), link to the review questions and objectives, and consider the relevance to key groups. | 20-21 |
| Limitations | 20 | Discuss the limitations of the scoping review process. | 25 |
| Conclusions | 21 | Provide a general interpretation of the results with respect to the review questions and objectives, as well as potential implications and/or next steps. | 26 |
| **FUNDING** | | | |
| Funding | 22 | Describe sources of funding for the included sources of evidence, as well as sources of funding for the scoping review. Describe the role of the funders of the scoping review. | 27 |

JBI = Joanna Briggs Institute; PRISMA-ScR = Preferred Reporting Items for Systematic reviews and Meta-Analyses extension for Scoping Reviews.

**Table S2.** Example Search Strategy for PubMed (MEDLINE) database.

| **Line** | **Search Terms** |
| --- | --- |
| 1 | ("Subsaharan Africa"[Title/Abstract] OR "Sub-Saharan Africa"[Title/Abstract] OR Angola[Title/Abstract] OR Benin[Title/Abstract] OR Botswana[Title/Abstract] OR "Burkina Faso"[Title/Abstract] OR Burundi[Title/Abstract] OR "Cabo Verdi"[Title/Abstract] OR Cameroon[Title/Abstract] OR "Cape Verde"[Title/Abstract] OR Central Africa*[Title/Abstract] OR Chad[Title/Abstract] OR Comoros[Title/Abstract] OR Congo[Title/Abstract] OR "Cote d'Ivoire"[Title/Abstract] OR Djibouti[Title/Abstract] OR "Equatorial Guinea"[Title/Abstract] OR Eritrea[Title/Abstract] OR Eswatini[Title/Abstract] OR Ethiopia[Title/Abstract] OR Gabon[Title/Abstract] OR Gambia[Title/Abstract] OR Ghana[Title/Abstract] OR Guinea[Title/Abstract] OR Guinea-Bissau[Title/Abstract] OR Kenya[Title/Abstract] OR Lesotho[Title/Abstract] OR Liberia[Title/Abstract] OR Madagascar[Title/Abstract] OR Malawi[Title/Abstract] OR Mali[Title/Abstract] OR Mauritania[Title/Abstract] OR Mayotte[Title/Abstract] OR Mauritius[Title/Abstract] OR Mozambique[Title/Abstract] OR Namibia[Title/Abstract] OR Niger[Title/Abstract] OR Nigeria[Title/Abstract] OR Reunion[Title/Abstract] OR Rwanda[Title/Abstract] OR "Sao Tome"[Title/Abstract] OR Sahel[Title/Abstract] OR Principe[Title/Abstract] OR Senegal[Title/Abstract] OR Seychelles[Title/Abstract] OR "Sierra Leone"[Title/Abstract] OR Somalia[Title/Abstract] OR "South Africa"[Title/Abstract] OR Sudan[Title/Abstract] OR Swaziland[Title/Abstract] OR Tanzania[Title/Abstract] OR Togo[Title/Abstract] OR Uganda[Title/Abstract] OR "Western Sahara"[Title/Abstract] OR Zambia[Title/Abstract] OR Zimbabwe[Title/Abstract] OR "Africa South of the Sahara"[MeSH]) |
| 2 | ("risk*"[Title/Abstract] OR "predict*"[Title/Abstract] OR "screen*"[Title/Abstract] OR "decision"[Title/Abstract]) AND ("model*"[Title/Abstract] OR "tool*"[Title/Abstract] OR "rule*"[Title/Abstract] OR "algorithm"[Title/Abstract] OR "scor*"[Title/Abstract] OR "calculat*"[Title/Abstract] OR "chart*"[Title/Abstract]) |
| 3 | ("Cardiovascular Diseases"[MeSH Terms] OR "Heart Disease Risk Factors"[MeSH Terms] OR "cardiovascular disease*"[Title/Abstract] OR "cardio-vascular disease*"[Title/Abstract] OR "heart disease*"[Title/Abstract] OR "vascular disease*"[Title/Abstract] OR "cardiovascular disorder*"[Title/Abstract] OR "cardio-vascular disorder*"[Title/Abstract] OR "heart disorder*"[Title/Abstract] OR "vascular disorder*"[Title/Abstract] OR "cvd"[Title/Abstract] OR "cardiovascular complication*"[Title/Abstract] OR "adverse cardiovascular event*"[Title/Abstract]) |
| 4 | 1 AND 2 AND 3 |

**Table S3.** Key Characteristics of Studies included in the Scoping Review.

| **Citation of study identified through main search** | **Year** | **Country** | **Study design** | **Population / setting** | **Risk score(s)** | **Study purpose** | **Summary of key findings** | **Use case(s) of risk score** |
| --- | --- | --- | --- | --- | --- | --- | --- | --- |
| Alwan H, William J, Viswanathan B, Paccaud F, Bovet P. Perception of cardiovascular risk and comparison with actual cardiovascular risk. Eur J Cardiovasc Prev Rehabil. 2009 Oct;16(5):556–61. | 2009 | Seychelles | Cross-sectional survey | Adults aged 40–64 years in a national survey (n=816) | WHO/ISH lab score (African region) | Assess how adults perceive their 10-year CVD risk and compare perceived risk with calculated WHO/ISH absolute risk | 59% could provide a semi-quantitative perceived risk estimate. 48% overestimated risk vs calculated risk. Ability to report perceived risk strongly associated with higher SES/education | Risk communication |
| Aw M, Ochieng BO, Attambo D, Opot D, Aw J, Francis S, et al. Critical appraisal of a mHealth-assisted community-based cardiovascular disease risk screening program in rural Kenya: an operational research study. Pathog Glob Health. 2020 Oct;114(7):379–87. | 2020 | Kenya | Implementation/ operational study | Convenience sample of 1650 adults ≥40 years screened by one CHW in rural Isiolo County over 24 months | WHO/ISH 10-year CVD risk chart (AFR E, no cholesterol) delivered via AFYACHAT SMS tool (4-tier risk) | Describe real-world implementation of AFYACHAT screening; acceptability, participant perceptions, and actions after high-risk results | One CHW screened 1650 people; among elevated risk participants, 75% sought further care; high acceptability, coding errors (15%), high participant satisfaction | Screening and referral |
| Basu S, Wagner RG, Sewpaul R, Reddy P, Davies J. Implications of scaling up cardiovascular disease treatment in South Africa: a microsimulation and cost-effectiveness analysis. Lancet Glob Health. 2019 Feb;7(2):e270–80. | 2019 | South Africa | Economic evaluation and modelling: microsimulation and cost-effectiveness evaluation | Simulated national population using SANHANES 2012 risk factor data; compares WHO PEN vs SA Primary Care 101 scale-up | WHO/ISH (lab), Gaziano (lab) | Assess distribution of risk factors and prioritise treatments; estimate DALYs and costs under expanded access scenarios | Scaling guideline-based treatment averts DALYs and can be cost-saving; suggests prioritising BP agents/statins | Economic and policy considerations |
| Bekele DM, Goshu DY, Yalew AW, Higgins MK, Gary RA. Low Subjective Cardiovascular Disease Risk Perceptions among Hypertensive Patients in Addis Ababa, Ethiopia. Integr Blood Press Control. 2022;15:81–96. | 2022 | Ethiopia | Institution-based cross-sectional study | Hypertensive patients in hospital follow-up clinics | Non-laboratory Framingham | Compare perceived vs objective CVD risk | Patients underestimated their CVD risk compared to calculated risk. | Risk communication |
| Beshah SA, Husain MJ, Dessie GA, Worku A, Negeri MG, Banigbe B, et al. Cost analysis of the WHO-HEARTS program for hypertension control and CVD prevention in primary health facilities in Ethiopia. Public Health Pract (Oxf). 2023 Dec;6:100423. | 2023 | Ethiopia | Economic evaluation: Programme cost analysis, micro-costing | Adult primary care users in catchment areas of 5 primary health facilities | CVD risk categories used for follow-up frequency (low/ medium/ high risk); scenario includes “integrated CVD risk management” package | Estimate health-system costs of implementing HEARTS under (1) hypertension-only and (2) integrated CVD risk management scenarios | Cost per adult primary care user: USD 5.3 (HTN control) vs USD 19.3 (integrated CVD risk management). Meds were major cost driver; in integrated scenario, lab diagnostics dominated (64%). | Economic and policy considerations |
| Gaziano T, Abrahams-Gessel S, Surka S, Sy S, Pandya A, Denman CA, et al. Cardiovascular Disease Screening By Community Health Workers Can Be Cost-Effective In Low-Resource Countries. Health Aff (Millwood). 2015 Sep;34(9):1538–45. | 2015 | South Africa | Economic evaluation: cost-effectiveness microsimulation | Hypothetical cohorts (adults 35–74) representing national populations | Gaziano non-laboratory CVD risk score | Estimate health and economic impact of shifting screening to CHWs using paper vs mobile tool | CHW screening was found to be cost-effective (often cost-saving); mobile app generally most cost-effective | Economic and policy considerations |
| Gaziano TA, Abrahams-Gessel S, Denman CA, Montano CM, Khanam M, Puoane T, et al. An assessment of community health workers’ ability to screen for cardiovascular disease risk with a simple, non-invasive risk assessment instrument in Bangladesh, Guatemala, Mexico, and South Africa: an observational study. Lancet Glob Health. 2015 Sep;3(9):e556-563. | 2015 | South Africa | Observational implementation study | Community adults aged 35–74 screened by CHWs | Gaziano non-laboratory CVD risk score | Assess agreement between CHW and professional risk scoring | High agreement achieved between CHW and health professional calculated scores; supporting feasibility of task-shifting of CVD risk screening to CHWs | Screening and referral |
| Gaziano TA, Steyn K, Cohen DJ, Weinstein MC, Opie LH. Cost-effectiveness analysis of hypertension guidelines in South Africa: absolute risk versus blood pressure level. Circulation. 2005 Dec 6;112(23):3569–76. | 2005 | South Africa | Economic evaluation: Cost-effectiveness model (Markov) | Adults without CVD (policy simulation) | Framingham (lab) | Compare absolute-risk vs blood pressure (BP)-threshold guidelines | Absolute-risk–based guidelines were more cost-effective than BP-based (single risk factor) approaches | Economic and policy considerations |
| Hamada Y, Mukora R, Pelusa R, Ntshiqa T, Shedrawy J, Velen K, et al. Costs and cost-effectiveness of integrated screening for non-communicable diseases in TB contacts. IJTLD Open. 2025 Mar;2(3):160–5. | 2025 | South Africa | Economic evaluation: Cross-sectional costing study and decision tree model over 10 years | TB household contacts screened for NCDs (hypertension, diabetes, CKD, dyslipidaemia) | WHO 2019 lab score (HEARTS package) | Estimate costs and cost-effectiveness of integrating NCD screening into TB contact investigation | Incremental cost USD 72.3/contact screened; but ICER USD 27,043.6/DALY averted, above SA threshold; not cost-effective mainly due to downstream care costs. | Economic considerations |
| Kamp M, Pain O, May A, Lewis CM, Ramsay M. Clinicians’ Perceptions towards Precision Medicine Tools for Cardiovascular Disease Risk Stratification in South Africa. Journal of Personalized Medicine. 2022 Sep;12(9):1360. | 2022 | South Africa | Cross-sectional survey | 109 medical doctors in four academic hospitals in Johannesburg South Africa | Precision medicine tools including polygenic risk scores (PRS) for CVD stratification | Explore clinicians’ existing use of CVD risk scores and perceptions of PM/PRS-based CVD risk stratification and barriers to adoption | 60% of clinicians using stratification tool to determine risk, with the Framingham Score being the most used (80%). Clinicians are confident tools with the majority sharing results with patients (86%) and using them to guide treatment (96%). | Screening and treatment |
| Kuti MA, Kuti KM, Awolude OA, Ogundeji OA, Moradeyo DM, Feinstein MJ, et al. Integration of Primary Preventive Care of Cardiovascular Disease in a Retroviral Clinic in an adult retroviral clinic in Ibadan: A retrospective study. Niger J Clin Pract. 2024 Sep 1;27(9):1082–8. | 2024 | Nigeria | Retrospective clinical audit | People living with HIV attending adult ART clinic | ASCVD PCE | Evaluate integration of ASCVD prevention in HIV care | Cardiovascular preventive practices were suboptimal; ASCVD risk was not calculated for any patient during their clinic visits. | Screening and treating high CVD risk |
| Levitt NS, Puoane T, Denman CA, Abrahams-Gessel S, Surka S, Mendoza C, et al. Referral outcomes of individuals identified at high risk of cardiovascular disease by community health workers in Bangladesh, Guatemala, Mexico, and South Africa. Glob Health Action. 2015;8:26318. | 2015 | South Africa | Observational implementation study | Community members identified as high risk by CHWs | Gaziano non-laboratory CVD risk score | Examine referral outcomes after risk CHW screening | Referral attendance was low (37%). Reasons for non-attendance included limited self-perception of risk, associated costs, health system obstacles, and lack of trust in CHWs to conduct CVD risk assessments and to refer community members into the health system. | Screening and referral |
| Mannik J, Figol A, Churchill V, Aw J, Francis S, Karino E, et al. Community-based screening for cardiovascular risk using a novel mHealth tool in rural Kenya. J Innov Health Inform. 2018 Oct 31;25(3):176–82. | 2018 | Kenya | Implementation/ operational study | CHW-led community screening in remote rural communities | WHO/ISH 10-year CVD risk chart (AFR E, non-lab) delivered via AFYACHAT SMS tool | Design and test SMS-based mHealth tool for CHWs to identify high CVD risk people needing education/pharmacologic intervention | Five CHWs screened 2,865 people over 22 months; tool operationalised (demonstrating feasibility). | Screening and referral |
| Melaku EE, Ayele ET, Urgie BM, Ayidagnuhim GB, Hassen EM, Tefera AS. Appropriate Use of Primary Statin Preventive Therapy Among Patients with High Atherosclerosis-Related Cardiovascular Disease Risks: Cross-Sectional Study, Northeast Ethiopia. Vasc Health Risk Manag. 2023;19:707–18. | 2023 | Ethiopia | Retrospective clinical audit and cross sectional survey | High-ASCVD-risk patients attending hospital follow-up clinic. | ASCVD Pooled Cohort Equations (PCE) | Assess magnitude of statin primary prevention use and associated factors among high-risk patients. | Statin primary prevention use was low (43%); use associated with diabetes duration, physician type (internists), regular follow-up, and higher 10-year ASCVD risk score | Treating high risk CVD |
| Mendis S, Abegunde D, Oladapo O, Celletti F, Nordet P. Barriers to management of cardiovascular risk in a low-resource setting using hypertension as an entry point. Journal of Hypertension. 2004;22:59–64. | 2004 | Nigeria | Cross-sectional assessment (facility survey, patient survey, provider interviews) | 56 randomly selected primary/secondary/ private facilities in Oyo State; 1000 consecutive known hypertensive patients attending follow-up | Absolute (total) CVD risk approach for hypertensive patients (implementation capacity assessed; no single named calculator) | Assess facility capacity and clinical practice patterns for implementing an absolute risk approach and effective CVD risk management in low-resource settings | Major barriers: limited lab/diagnostic availability (esp. primary care), knowledge gaps among patients/providers, non–evidence-based prescribing, and high out-of-pocket costs (73%). | Screening and treating high risk |
| Mosepele M, Letsatsi V, Mokgatlhe L, Hudson FP, Gross R. Cholesterol Screening and Statin Prescription is Low Among HIV-Infected Patients on Protease-Inhibitor Regimens in Botswana. Open AIDS J. 2017;11:45–51. | 2017 | Botswana | Retrospective clinical audit | HIV-infected adults on protease-inhibitor regimens | ASCVD PCE; Framingham (lab) | Assess cholesterol screening and statin prescribing gaps | Many patients were eligible for statins but very few (<1%) received them | Screening and treatment |
| Mphekgwana PM, Monyeki KD, Mothiba TM, Makgahlela M, Kgatla N, Malema RN, et al. Screening and Interventions for Cardiovascular Disease Prevention in the Limpopo Province, South Africa: Use of the Community Action Model. Metabolites. 2022 Nov 4;12(11):1067. | 2022 | South Africa | Implementation study (community-based referral intervention) | Rural communities (Capricorn district, Limpopo) | INTERHEART Non-laboratory | Screen using and develop/implement community-tailored intervention strategies | Reported significant pre–post improvements for some outcomes (e.g., raised SBP, BMI/waist circumference) and concludes CAM is feasible/effective in rural communities | Screening and referral |
| Muthee TB. Examining the Prevention and Management of Cardiovascular Disease Risk in Kenyan Primary Care Settings [Internet] [Ph.D.]. PQDT - UK & Ireland. [England]: University of Oxford (United Kingdom); 2022. Available from: <https://www.proquest.com/dissertations-theses/examining-prevention-management-cardiovascular/docview/2773485824/se-2?accountid=13042> | 2022 | Kenya | Qualitative case study using observations, interviews, patient exit interviews + document review (DPhil (PhD) thesis) | Kilifi County primary care facilities (levels 2–4); clinicians, patients (≥30 years), and policymakers | No specific score – focus on adoption of recommended CVD risk assessment practice | Examine how CVD risk prevention/management is practiced and identify contextual factors influencing adoption of recommended risk assessment in Kenyan primary care | Recommended practice was infrequently followed; clinicians relied on judgement due to limited guideline familiarity; identifies multi-level barriers | Screening and treatment |
| Mwita CC, Akello W, Sisenda G, Ogoti E, Tivey D, Munn Z, et al. Assessment of cardiovascular risk and target organ damage among adult patients with primary hypertension in Thika Level 5 Hospital, Kenya: a criteria-based clinical audit. Int J Evid Based Healthc. 2013 Jun;11(2):115–20. | 2013 | Kenya | Retrospective clinical audit | Adults with hypertension attending medical outpatient clinic, Thika Level 5 Hospital | No specific score | Assess compliance with guidelines for CVD risk | No CVD risk tool use was documented in the audits. High patient load was identified as the biggest barrier to implementation. | Screening and treatment of high risk |
| Ndede KO, Khan Z, Akumiah FK, Wanyoike M. A Retrospective Five-Year Study of Cardiovascular Risk Assessment and Risk-Based Interventions Among Hypertensive Patients in Nairobi Hospital, Kenya. Cureus. 15(9):e46097. | 2023 | Kenya | Retrospective clinical audit | 373 hypertensive patient records from a private cardiology clinic (Nairobi Hospital) | Guideline-recommended WHO/ISH East African risk charts (lab or non-lab) and any alternative tools; documentation of 10-year CVD risk | Audit clinician use of CVD risk assessment tools and whether primary prevention interventions are risk-based | Very low documented use (1.3%) of national WHO/ISH charts and risk documentation; despite this, many patients received lifestyle advice and about half were on statins for primary prevention | Screening and treatment |
| Ndindjock R, Gedeon J, Mendis S, Paccaud F, Bovet P. Potential impact of single-risk-factor versus total risk management for the prevention of cardiovascular events in Seychelles. Bull World Health Organ. 2011 Apr 1;89(4):286–95. | 2011 | Seychelles | Economic evaluation: Comparative modelling / policy analysis | Adults 40–64 from national survey | WHO/ISH | Compare single-risk-factor treatment vs total-risk-based management (≥10% or ≥20% 10-year risk) in terms of people treated and events averted; assess relative cost-effectiveness (medication costs) | Total-risk management treated far fewer people while still averting substantial events; authors conclude total CVD risk management is much more cost-effective than single-risk-factor management | Economic and policy considerations |
| Ngalesoni FN, Ruhago GM, Mori AT, Robberstad B, Norheim OF. Cost-effectiveness of medical primary prevention strategies to reduce absolute risk of cardiovascular disease in Tanzania: a Markov modelling study. BMC Health Serv Res. 2016 May 17;16(1):185. | 2016 | Tanzania | Economic evaluation: cost-effectiveness analysis using Markov models | Hypothetical cohorts representing four WHO absolute-risk levels (with and without diabetes) | WHO/ISH lab | Estimate cost-effectiveness of medical primary prevention strategies by absolute CVD risk level | Preventive drug combinations were “very cost-effective” for most risk levels except low risk; thresholds/ICERs varied by risk group and diabetes status | Economic and policy considerations |
| Ngalesoni FN, Ruhago GM, Mori AT, Robberstad B, Norheim OF. Equity impact analysis of medical approaches to cardiovascular diseases prevention in Tanzania. Soc Sci Med. 2016 Dec;170:208–17. | 2016 | Tanzania | Economic evaluation: Economic & equity modelling study | Hypothetical national adult cohorts | WHO/ISH lab; ESC SCORE and age-differentiated thresholds | Assess efficiency and equity of alternative CVD prevention guidelines | Age-differentiated risk thresholds improve both efficiency and equity | Economic and policy considerations |
| Niyibizi JB, Ntawuyirushintege S, Nganabashaka JP, et al. Community Health Worker-Led Cardiovascular Disease Risk Screening and Referral for Care and Further Management in Rural and Urban Communities in Rwanda. Int J Environ Res Public Health. 2023;20:5641. | 2023 | Rwanda | Action research / implementation study | Community adults aged 35–74 in rural and urban communities, screened by CHWs | Non-laboratory Framingham (BMI-based) score | Assess feasibility and accuracy of CHW-led CVD risk screening and referral | CHWs accurately assessed CVD risk (good correlation with nurses); Referral uptake moderate (~40–70%), lower in urban areas; Barriers: cost, time, low risk perception | Screening and referral |
| Odunaiya NA, Adegoke OM, Adeoye AM, Oguntibeju OO. Preliminary study of perceived cardiovascular disease risk and risk status of adults in small rural and urban locations in Ibadan, Nigeria. AIMS Public Health. 2023;10(1):190–208. | 2023 | Nigeria | Mixed-methods: cross-sectional survey and exploratory qualitative focus group discussions | Community adults (rural + urban) with no diagnosed CVD at baseline | INTERHEART Non-laboratory | Compare actual CVD risk status vs perceived risk in rural vs urban groups | Risk perception was often low/misaligned; highlighting need for education/ prevention | Risk communication |
| Puoane T, Abrahams-Gessel S, Gaziano TA, Levitt N. Training community health workers to screen for cardiovascular disease risk in the community: experiences from Cape Town, South Africa. Cardiovasc J Afr. 2017;28(3):170–5. | 2017 | South Africa | Training evaluation of implementation | CHWs screen households and refer to facilities for validation | Gaziano non-laboratory CVD risk score | Describe CHW training process and assess learning/feasibility of CHWs performing community CVD risk screening | Training improved knowledge; CHWs were able to screen/identify at-risk people and refer, but training duration and language of instruction/testing were challenges | Screening and referral |
| Rosendaal NTA, Hendriks ME, Verhagen MD, Bolarinwa OA, Sanya EO, Kolo PM, et al. Costs and Cost-Effectiveness of Hypertension Screening and Treatment in Adults with Hypertension in Rural Nigeria in the Context of a Health Insurance Program. PLoS One. 2016;11(6):e0157925. | 2016 | Nigeria | Economic evaluation: Cost-effectiveness model (Markov) | Rural adults within health insurance programme (Kwara State) | Framingham (lab) | Evaluate cost-effectiveness of hypertension screening and treatment | Screening and treatment targeted to high-risk individuals may be cost-effective, though affordability remains uncertain | Economic considerations |
| Sando D, Kintu A, Okello S, Kawungezi PC, Guwatudde D, Mutungi G, et al. Cost-effectiveness analysis of integrating screening and treatment of selected non-communicable diseases into HIV/AIDS treatment in Uganda. J Int AIDS Soc. 2020 Jun;23 Suppl 1(Suppl 1):e25507. | 2020 | Uganda | Economic evaluation: Economic decision-analytic model | Adults living with HIV on ART (national cohort) | Globorisk | Assess cost-effectiveness of integrating NCD screening into HIV care | Integrated NCD screening could reduce CVD risk and be cost-effective, especially in older PLWH | Economic considerations |
| Sarfo FS, Akinyemi JO, Obiako R, Nichols M, Fakunle AG, Adusei N, et al. Effect of an Educational Intervention for Primary Stroke Risk Reduction in Ghana and Nigeria: Pilot Randomized Controlled Trial. Stroke. 2023 Jun;54(6):1660–4. | 2023 | Ghana, Nigeria | Pilot randomised controlled trial | Adults with multiple vascular risk factors in urban/peri-urban clinical & community settings | Afrocentric stroke risk score | Test feasibility of an mHealth intervention using a risk score for primary stroke prevention | mHealth education plus Afrocentric risk tool was feasible and showed a positive signal for reducing overall stroke risk and improving BP over 2 months | Screening and referral and communicating risk |
| Sarfo FS, Obiako R, Nichols M, Akinyemi JO, Fakunle A, Akpa O, et al. Knowledge and perspectives of community members on risk assessment for stroke prevention using mobile health approaches in Nigeria. J Stroke Cerebrovasc Dis. 2023 Sep;32(9):107265. | 2023 | Nigeria | Cross-sectional survey | Community-dwelling, stroke-free adults in Ibadan, Abeokuta, Zaria | Afrocentric stroke risk score | Assess knowledge, preferences, and willingness to use mHealth stroke risk assessment | Community members expressed high desire to know immediate stroke risk. Cobile risk calculators were acceptable. | Communicating risk |
| Ssekubugu R, Makumbi F, Enriquez R, Lagerström SR, Yeh PT, Kennedy CE, et al. Cardiovascular (Framingham) and type II diabetes (Finnish Diabetes) risk scores: a qualitative study of local knowledge of diet, physical activity and body measurements in rural Rakai, Uganda. BMC Public Health. 2022 Nov 29;22(1):2214. | 2022 | Uganda | Qualitative study:  Semi-structured interviews | Purposive sample (n=15) from rural Uganda | Framingham Risk Score (non-lab) | Assess local understanding/terminology and acceptability of key risk-score components (diet, physical activity, anthropometrics, family history) in rural Uganda | Terms related to CVD risk varied in their acceptability among respondents. While the risk scores are generally suitable, it is important to localize key aspects, especially physical activity and taking seasonality into account for fruit and vegetable consumption | Risk communication |
| Subramanian S, Hilscher R, Gakunga R, Munoz B, Ogola E. Cost-effectiveness of risk stratified medication management for reducing premature cardiovascular mortality in Kenya. PLOS ONE. 2019 Jun 25;14(6):e0218256. | 2019 | Kenya | Economic evaluation: Economic microsimulation model | Simulated national adult population (25–69 years) | Framingham (lab) | Assess cost-effectiveness of risk-stratified hypertension treatment | Risk-based treatment targeting high-risk groups can be cost-effective under low drug-cost scenarios. The results are highly sensitive to cost of medications. | Economic considerations |
| Surka S, Edirippulige S, Steyn K, Gaziano T, Puoane T, Levitt N. Evaluating the use of mobile phone technology to enhance cardiovascular disease screening by community health workers. Int J Med Inform. 2014 Sep;83(9):648–54. | 2014 | South Africa | Mixed methods pilot study implementation and feasibility study | Community health workers (n=24) screening community members (n=537) using paper vs mobile tool | Non-laboratory CVD absolute risk tool (Gaziano et al. model) | Develop and evaluate a mobile CVD risk app and compare impact on CHW training time, screening time, and calculation errors vs paper charts | Mobile app reduced training time (3h vs 12.3h), reduced screening time (21 vs 36 min), and eliminated calculation errors; high end-user satisfaction | Screening and referral |
| Surka S, Steyn K, Everett-Murphy K, Gaziano TA, Levitt N. Knowledge and perceptions of risk for cardiovascular disease: Findings of a qualitative investigation from a low-income peri-urban community in the Western Cape, South Africa. Afr J Prim Health Care Fam Med. 2015 Oct 22;7(1):891. | 2015 | South Africa | Qualitative study (focus group discussions) exploring risk understanding and presentation methods | Low-income peri-urban community members in Nyanga township; adults ≥25 years | Non-laboratory CVD absolute risk tool (Gaziano et al. model) | Explore understanding and perception of CVD risk and how risk is understood and best presented | Knowledge of CVD terms varied, but understanding of “risk” was poor; risk perceived as binary, making evaluation of risk-communication formats difficult | Communicating risk |
| Tolla MT, Norheim OF, Memirie ST, Abdisa SG, Ababulgu A, Jerene D, et al. Prevention and treatment of cardiovascular disease in Ethiopia: a cost-effectiveness analysis. Cost Eff Resour Alloc. 2016;14:10. | 2016 | Ethiopia | Economic evaluation: Cost-effectiveness analysis | Ethiopian population; evaluates prevention, acute treatment & secondary prevention strategies for IHD and stroke | WHO/ISH laboratory | Identify the most cost-effective CVD prevention/treatment interventions for scale-up in Ethiopia | Risk-based combination drug treatment at >35% 10-year risk was most cost-effective. Integrated acute/prevention packages improved efficiency when compared to single risk factor interventions | Economic and policy considerations |
| van de Vijver S, Oti S, Tervaert TC, Hankins C, Kyobutungi C, Gomez GB, et al. Introducing a model of cardiovascular prevention in Nairobi’s slums by integrating a public health and private-sector approach: the SCALE-UP study. Glob Health Action. 2013 Oct 21;6:22510. | 2013 | Kenya | Programme design for care pathway | Nairobi slum setting; community/home-based screening and linkage-to-care model | No specific score | Develop and introduce a feasible, potentially cost-effective CVD prevention service package in slums | Projected low per-person cost and “potentially cost effective” with use of performance based financing strategy | Screening and referral |
| **Additional studies identified through citation searches** | **Year** | **Country** | **Study design** | **Population / setting** | **Risk score(s)** | **Study purpose** | **Summary of key findings** | **Use case(s) of risk score** |
| Abrahams-Gessel S, Denman CA, Montano CM, Gaziano TA, Levitt N, Rivera-Andrade A, et al. Lessons from Training and Supervision of Community Health Workers conducting non-invasive, population-based screening for Cardiovascular Disease in Four Communities in Low and Middle-Income Settings: Implications for Scaling Up. Glob Heart. 2015 Mar;10(1):39–44. | 2015 | South Africa | Mixed-methods | Community adults screened by trained CHWs | Gaziano non-laboratory CVD risk score | Evaluate training and field performance of CHWs in CVD screening | CHWs can be trained quickly to conduct accurate non-invasive CVD risk screening | Screening and referral |
| Abrahams-Gessel S, Denman CA, Montano CM, Gaziano TA, Levitt N, Rivera-Andrade A, et al. The training and fieldwork experiences of community health workers conducting population-based, noninvasive screening for CVD in LMIC. Glob Heart. 2015 Mar;10(1):45–54. | 2015 | South Africa | Mixed-methods training & implementation evaluation (pre/post tests + FGDs on fieldwork experience) | CHWs trained to screen adults aged 35–74 years in communities served by local health centres; CHWs deployed for community/home screenings and referrals | Gaziano non-laboratory CVD risk score | Quantitatively assess CHW knowledge/skills acquisition and qualitatively capture CHW experiences implementing population-based CVD risk screening | Training improved CVD knowledge and was largely retained; key implementation themes included language/numeracy challenges (BMI), need for longer training, role clarity, respect/compensation, and strong community/context tailoring | Screening and referral |
| Kedir K, Okop K, Ayele FM, Getachew H, Dereje M, Hailu B, et al. High rates of voluntary clinic attendance among community members screened with high cardiovascular disease risk scores in the rural and urban communities of Adama, Ethiopia. BMC Public Health. 2025 Oct 22;25(1):3566. | 2025 | Ethiopia | Implementation study (community-based referral intervention) | 772 community members aged ≥35 years in 10 kebeles across rural Adama district, Adama town, and Addis Ababa | Framingham 10-year CVD risk algorithm embedded in a mobile app (REDCap-based) | Identify and refer moderate/high-risk individuals (>10% 10-year risk), assess clinic attendance/referral completion, and explore reasons for non-attendance (rural vs urban) | Among participants identified as high risk, 64.5% attended clinic (higher in rural vs urban); main non-attendance reasons were inconvenience, “feeling fine”, and financial challenges. | Screening and referral |
| Niyibizi JB, Okop KJ, Nganabashaka JP, Umwali G, Rulisa S, Ntawuyirushintege S, et al. Perceived cardiovascular disease risk and tailored communication strategies among rural and urban community dwellers in Rwanda: a qualitative study. BMC Public Health. 2022 May 9;22(1):920. | 2022 | Rwanda | Qualitative study with focus group discussions | Rural and urban community members (18–65 years; limited education) | No specific score – focus on how risk is presented | Explore perceptions of CVD risk and preferences for communicating estimated total CVD risk | Participants struggled with quantitative risk formats; preferred qualitative/colour formats and in-person explanation | Risk communication |
| Ofori S, Wachukwu C. Awareness and attitudes towards total cardiovascular disease risk assessment in clinical practice among physicians in Southern Nigeria. The Nigerian Health Journal [Internet]. 2016 Dec 13 [cited 2025 Feb 12];16(1). Available from: <https://www.ajol.info/index.php/nhj/article/view/149470> | 2016 | Nigeria | Cross-sectional survey | Physicians in government hospitals and private practices, Port Harcourt | No specific score | Assess awareness, use, and attitudes toward total CVD risk assessment in routine practice | Awareness of risk scoring was fairly high but regular use was low; unfamiliarity with how to use tools was a key barrier. | Treating high risk |

**Figure S1.** Adapted Implementation Research Logic Model (IRLM) showing all determinants, strategies and outcomes of implementation of cardiovascular disease risk prediction scores in Sub-Saharan Africa. Mechanisms were not clearly reported in the available literature. Key: (+) facilitator; (-) barrier; (+/-) facilitator or barrier depending on context. CFIR: Consolidated Framework for Implementation Research | ERIC: Expert Recommendations for Implementing Change | CVD: Cardiovascular Disease | CHW: Community Health Worker | BMI: Body Mass Index.
